# Specific and Sensitive Detection of *H. pylori* in Biological Specimens by Real-Time RT-PCR and In Situ Hybridization

**DOI:** 10.1371/journal.pone.0002689

**Published:** 2008-07-16

**Authors:** Hui Liu, Arifur Rahman, Cristina Semino-Mora, Sonia Q. Doi, Andre Dubois

**Affiliations:** Department of Medicine, Uniformed Services University of the Health Sciences, Bethesda, Maryland, United States of America; Baylor College of Medicine, United States of America

## Abstract

PCR detection of *H. pylori* in biological specimens is rendered difficult by the extensive polymorphism of *H. pylori* genes and the suppressed expression of some genes in many strains. The goal of the present study was to (1) define a domain of the *16S rRNA* sequence that is both highly conserved among *H. pylori* strains and also specific to the species, and (2) to develop and validate specific and sensitive molecular methods for the detection of *H. pylori*. We used a combination of *in silico* and molecular approaches to achieve sensitive and specific detection of *H. pylori* in biologic media. We sequenced two isolates from patients living in different continents and demonstrated that a 546-bp domain of the *H. pylori 16S rRNA* sequence was conserved in those strains and in published sequences. Within this conserved sequence, we defined a 229-bp domain that is 100% homologous in most *H. pylori* strains available in GenBank and also is specific for *H. pylori*. This sub-domain was then used to design (1) a set of high quality RT-PCR primers and probe that encompassed a 76-bp sequence and included at least two mismatches with other *Helicobacter sp. 16S rRNA*; and (2) *in situ* hybridization antisense probes. The sensitivity and specificity of the approaches were then demonstrated by using gastric biopsy specimens from patients and rhesus monkeys. This *H. pylori*-specific region of the *16S rRNA* sequence is highly conserved among most *H. pylori* strains and allows specific detection, identification, and quantification of this bacterium in biological specimens.

## Introduction

*Helicobacter pylori* is responsible for most duodenal and peptic ulcer and also plays an important role in gastric adenocarcinoma [1]–[3]. The mechanism of *H. pylori* pathogenic effect is unclear, but it is believed to be related to complex host bacterial interactions triggered by virulence genes [4], and it is possible that these effects are enhanced by the invasiveness of the bacterium [5]–[7]. Finally, *H. pylori* was recently observed within gastric mucosa capillaries, where it appears to establish close association with erythrocytes [7], [8]. Therefore, it is important to develop specific and sensitive molecular methods allowing the detection and identification of this microorganism in biological specimens.

Culture of the bacterium is considered the gold standard, but the method is not sensitive and is specific only if additional testing is performed on the isolates. The method of choice involves polymerase chain reaction (PCR) amplification of specific *H. pylori* genes. However, using this approach may be problematic due to the extensive polymorphism of many *H. pylori* genes and the absence of particular genes in some strains [e.g. cagA [9]]. Among the genes that have been tested, *ureA* and *ureC* (also named *glmM*) appear sensitive, but they lack specificity. Therefore, the concurrent detection of multiple, *H. pylori-*specific, genes and the use of different sets of primers has been considered to be necessary to achieve specific and sensitive diagnosis of the infection.

Another approach to the question has been to use *H. pylori 16S rRNA*. This ribosomal gene is particular in that it is present in all bacteria while, at the same time, it comprises nucleotide sequences that are specific to a given bacterial genus [10], [11]. Sequence analysis of the *16S rRNA* gene has led to our current understanding of prokaryotic phylogeny and *H. pylori 16S rRNA* gene sequence analysis unambiguously differentiated the *Helicobacter* genus from the closely related *Campylobacter* genus [12] thus allowing creation of the *Helicobacter* genus. Finally, *H. pylori 16S rRNA* gene sequence has been used as a tool to differentiate *H. pylori* from other *Helicobacter* sp. especially for isolates from animal sources [13]–[16].

Here, we sequenced the *16S rRNA* genes of two *H. pylori* strains with markedly different DNA fingerprints that had been cultured from two patients living in different continents and with different endoscopic diagnosis. By matching these sequences with each other and with those available in the National Center for Biotechnology Information (NCBI) nucleotide database, we first identified a unique nucleotide domain that is homologous in most *H. pylori* strains. We then defined, within this domain, a sequence that is homologous among *H. pylori* strains but not among other bacterial species and used this domain to design *H. pylori*-specific primers and probes to be used in a real-time quantitative RT-PCR (TaqMan) assay and an *in situ* hybridization (ISH) method. These methods can specifically detect less than 10 copies of *H. pylori* in gastric biopsies and also allow quantification of *H. pylori* density in biopsies from animals and patients with gastritis, gastric precancerous lesions and cancer.

## Methods

**Ethical approval** to carry studies in humans was obtained from Institutional Review Board of the participating institutions and written consent forms was obtained from each participant. In addition, studies performed in animals were approved by the Institutional Animal Care and Use Committee.

### *H. pylori* strains

Gastric antral biopsies were harvested in (1) an Albanian patient with gastric adenocarcinoma and (2) a U.S. Caucasian patient with marked gastritis but no ulcer. Biopsies were cultured using *Campylobacter* chocolatized blood agar plates supplemented with Trimethoprim, Vancomycin, Amphotericin B and Polymyxin B (Remel, Lenexa) at 37°C in an atmosphere of 90% N_2_, 5% O_2_, and 5% CO_2_ (microaerobic conditions). Bacterial isolates consistent with *H. pylori* in shape, colony morphology, enzymatic activity, and Gram-negative status grew within 7–10 days. Single colony isolates were subcultured on sheep blood agar plates supplemented with Tryptic Soy Agar (Remel, Lenexa, KS), confirmed for enzymatic activity and Gram stain and collected in phosphate buffer saline (PBS; 137 mM NaCl, 2.7 mM KCl, 10 mM phosphate buffer) for subsequent genomic DNA extraction and analysis.

### DNA extraction

DNA was extracted from each isolate collected in PBS by QIAamp DNA mini kit and processed the samples as described in the insert (Qiagen Inc., Stanford).

### Random Amplification of Polymorphic DNA (RAPD)

DNA fingerprinting using the RAPD technique was used to compare the isolates. A set of 5 different 10-mer primers (1247: 5′-AAGAGCCCGT-3′; 1254: 5′-CCGCAGCCAA-3′; 1281: 5′-AACGCGCAAC-3′; 1238 5′- GCGATCCCCA-3′; 1290: 5′- GTGGATGCGA-3′) were used as published [17].

### *16S rRNA* gene amplification and sequencing

Total DNA was extracted from each patient's isolate and PCR-amplified using published primers (see supplementary Methods S1) [18]. The Basic Local Alignment Search Tool (nucleotide BLAST), National Center for Biotechnology Information (NCBI), NIH, (http://www.ncbi.nlm.nih.gov/blast/Blast.cgi) feature for alignment between two nucleotide sequences (bl2seq) [19] was used to align the overlapping sequenced segments of the *16S rRNA* gene. The *16S rRNA* sequences of strains USU101 and USU102 were decoded and registered in the GenBank nucleotide database as EU544199 and EU544200, respectively.

### Histology and *in situ* hybridization

Gastric biopsies were fixed in 4% paraformaldehyde within 30 seconds of harvesting, dehydrated in ethanol within two days, and embedded in paraffin. Unstained sections were then stained with hematoxylin and eosin or according to Genta [20] or processed for ISH as described in the supplementaries [5], [21].

### Controls of method

Control for nonspecific binding was performed by using: (1) sense instead of antisense probe; (2) hybridization buffer instead of antisense probe; (3) unlabeled antisense probe; (4) digoxigenin or biotin-labeled probe for scorpion Butus martensi Karsch neurotoxin sequence [5′-GGC CAC GCG TCG ACT AGT AC-3′] [22]; (5) RNaseA pretreatment (Roche); (6) DNase I pretreatment (Roche); and (7) RNase plus DNase I pretreatment.

### *In silico* search for a *16S rRNA* sequence conserved in, and specific for, *H. pylori* strains

The DNASTAR software (www.dnastar.com) was used to perform multi-alignment of the two decoded sequences described above along with the sequences of the three strains that have been completely sequenced to date (J99, 26695, and HPAG1) and with the published sequences of the *16S* ribosomal RNA of *E. coli* (J01859), *S. bareilly* (U92196), *C. jejuni* (LO4315), *S. flexneri* (AE016991 AE014073), and *H. heilmannii* (AF506793).

### Design of primers and probes specifically recognizing published *H. pylori* strains

The PrimerExpress® v2.0 Software was used to design multiple sets of real-time RT-PCR primers flanking an oligonucleotide probe. The rules and requirements described in the PrimerExpress tutorial [23] were then applied to select the set that would provide maximum sensitivity and specificity of the assay. Locus-specific primers flanking an oligonucleotide probe labeled with a 5′ fluorescent Reporter dye (FAM or TET) and a 3′ Quencher dye (TAMRA) were ordered from Applied Biosystems (www.appliedbiosystems.com).

### Validation of the primers and probes

Pure cultures of *H. pylori*, *E. coli* (Top10, Invitrogen, Carsbad, CA), *S. typhimurium* LT2, *V. cholerae* O139 (Classical Ogawa), *V. cholerae* O139 (El Tor), and *P. aeruginosa* were lysed and total DNA was extracted. The specificity of the primers and probes described above was then verified by real-time PCR using an ABI PRISM 7500 Sequence Detection System (Applied Biosystems) [24].

In addition, smears of the pure cultures were streaked onto glass slides, immediately covered with a drop of 4% paraformaldehyde, and let to dry overnight. The next day, they were processed for ISH as described above.

### Cloning of the standard cRNA

The MEGAscript protocol for Standard cRNA cloning (MEGAscript high yield transcription kit, Ambion) was used to incorporate the SP6 promoter into *H. pylori* strain J99 *16S rRNA* at a location situated upstream of the sequence of interest, thus ensuring that the promoter sequence was incorporated into the PCR product. Conditions for primer extension were 95°C for 15 sec, 60°C for 15 sec, 72°C for 1 min. for 38 cycles to produce a 246 bp PCR product. The ABI Prism BigDye Terminator Cycle Sequencing Ready Reaction kit was used to verify that the sequence of the PCR product was identical to the corresponding *16S rRNA* sequence. In vitro transcription of cRNA was then performed using 2 µL (0.2 µg) of the PCR product as a template with the MEGAscript High Yield Transcription Kit (Ambion). This reverse transcription product was purified by RNeasy Mini Kit and treated with DNaseI during this purification (Qiagen). The concentration of this cRNA was calculated from the mean of three OD measurements and then converted to the copy numbers using Avogadro's number. The stock solution was aliquoted from freshly prepared 10-fold serial dilutions from 10^1^ to 10^6^ copies and stored at −80°C.

### Absolute quantitative real-time RT-PCR (QRT-PCR)

A single-tube reaction with a TaqMan One-Step RT-PCR Master Mix Reagents kit (Applied Biosystems) designed for reverse transcription (RT) and polymerase chain reaction (PCR) in a single buffer system was used in an ABI PRISM 7500 Sequence Detection System (Applied Biosystems, Foster City, CA). The primers and probes concentrations were first optimized using controls from a pool of total RNA extracted from *H. pylori* cultures and monkey gastric biopsies (BioChain Institute, Inc. Hayward, CA). The assay was then performed by adding 2 µl of 50 ng/µl monkey total RNA aliquots to the real time RT-PCR reaction mix to a final volume of 50 µl. The RT step was performed at 48°C for 30 min, followed by 10 min at 95°C for AmpliTaq Gold Activation. The PCR step consisted of 40 cycles of denature 15 sec at 95°C and anneal/extend 1 min at 60°C. All samples and cRNA standards were assayed without reverse transcriptase to confirm the absence of DNA contamination.

Conversion of Ct values to *H. pylori 16S rRNA* copy numbers was performed using linear regression analysis of a standard curve derived from serial 10^1^ to 10^6^ copies, 10-fold dilutions of the cloned cRNA.

### Gastric biopsies

Three biopsies were obtained from each of the 23 rhesus monkeys studied in an inoculation experiment [25]. As described above, the first biopsy was cultured for *H. pylori*, the second biopsy was fixed in formalin and either stained according to Genta [20] or unstained sections were processed for ISH, and the third biopsy was processed to extract total RNA.

### Statistical Analysis

Data were entered into our Microsoft Access database. Log-transformed copy numbers were normally distributed. Pearson correlation coefficients (r) and associated probabilities (P) were calculated and a two-sided P-value of 0.05 or less was considered statistically significant.

## Results

### DNA fingerprinting

**Figure 1:**
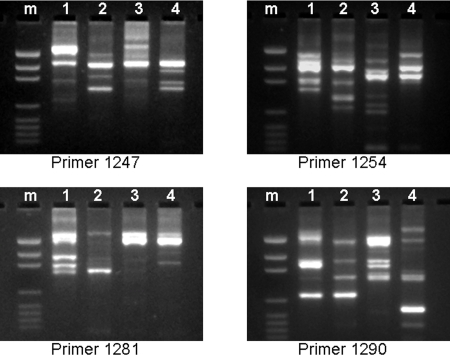
DNA fingerprinting (RAPD) of four *H. pylori* strains: USU101, isolated from an Albanian patient with gastric adenocarcinoma (1), USU102, isolated from a U.S. Caucasian patient with no ulcer (2), strain J99 (3), and strain 26695 (4). Note that the DNA fingerprints of the four strains are quite different from each other.

In order to study the genomic diversity between various *H. pylori* strains, we performed RAPD fingerprinting analysis of strains USU101, USU102, J99, and 26695. As shown in Figure 1, the pattern of these four strains was markedly different from each other in regard to all 4 primers used for RAPD.

### *In silico* search for a *16S rRNA* sequence conserved in *H. pylori* strains

**Figure 2:**
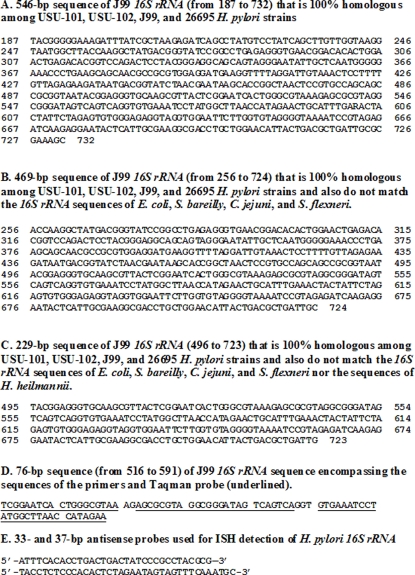
Sequences of *H. pylori 16S rRNA* that are 100% homologous among USU-101, USU-102, J99, and 26695 *H. pylori* strains (A), and also do not match the *16S rRNA* sequences of *E. coli*, *S. bareilly*, *C. jejuni*, and *S. flexneri* (B), nor the sequences of *H. heilmannii* (C), and encompass the set of primers and TaqMan probe (D). E shows the sequences of the two ISH probes used in the present study (546-bp from 187 to 732 of J99 *16S rRNA* sequence).

To examine whether a particular domain of *H. pylori 16S rRNA* sequence was conserved among strains with markedly different fingerprints, the DNASTAR software was used to perform multi-alignment of the *16S rRNA* sequences of the four strains described above**.** We discovered that a 546-bp nucleotide domain was 100% conserved among these five sequences (Figure 2A). To determine whether this domain was also conserved among various *H. pylori* strains, we performed a nucleotide BLAST of this sequence and observed that the sequence was 100% homologous to 49 *H. pylori* sequences published in GenBank to date.

### *In silico* search for a conserved *16S rRNA* sequence that is also specific to *H. pylori* strains

In order to search for a region that is specific for *H. pylori*, the conserved 546-bp nucleotide domain was entered into the DNAStar software along with the published *16S rRNA* sequences of *E. coli*, *S. bareilly*, *C. jejuni*, *S. flexneri*, and *H. heilmannii*. We observed that a 229-bp domain of the conserved region did not match the other five bacteria (Figure 2C). Basic nucleotide BLAST alignment (Blastn) of this sequence demonstrated complete homology with 74 *H. pylori* strains, two *H. nemestrinae* and four *Helicobacter* sp. “liver” (that were subsequently found to be indistinguishable from *H. pylori* [26], [27]), and 17 uncultured *Helicobacter* species. Sequences of these uncultured *Helicobacter* species had been determined from biopsies from human esophageal carcinoma or inflamed colon [28], from the stomach of cheetahs [a carnivore that is frequently colonized by the closest *H. pylori* relative, *H. acinonychis* [29]], or from the stomach of thoroughbred horses [30].

The following TaqMan RT-PCR primers and probe were then designed within the 229-bp sequence as described in Materials and Methods: forward primer 5′-TCG GAA TCA CTG GGC GTA A-3′; reverse primer 5′-TTC TAT GGT TAA GCC ATA GGA TTT CAC-3′; probe 5′–TGA CTG ACT ATC CCG CCT ACG CGC T-3′ (Figure 2D).

In addition, two probes for in situ hybridization (ISH) were designed within the same 229-bp sequence (Figure 2E).

### *In silico* validation of the RT-PCR set of primers and probe and of the ISH probes

In order to validate the specificity of the set of two primers and a probe used in our real-time RT-PCR assay, we performed a BLAST alignment of the corresponding 76-bp sequence (Figure 2D) with the GenBank database. We observed 100% homology with 136 *H. pylori* strains, three *H. nemestrinae* and four *Helicobacter* sp. “liver” (that are, in fact, *H. pylori* [26], [27]), one *H. acinonychis* [29] and 37 uncultured *Helicobacter* species (isolated from human esophageal carcinoma, inflamed colon, or liver [28], [31], from seven cheetahs, and from a tiger). In addition, two *H. pylori* 16S RNA sequences (AY057935 and AY057936) showing a low homology (91% and 97%, respectively) with the 76-bp nucleotide sequence were isolates referred to the genomic sequences of the *H. pylori* strains 26695 and J99 in the ATCC catalog. It is noteworthy, however, that in contrast to these two ATCC isolates, both 26695 and J99 strains are among those showing 100% homology with our 76-bp sequence. To clarify this apparent discrepancy, we performed BLAST alignment of AY057935 and AY057936 with their respective parental strains, and found 82 and 91% homology, respectively. Thus, it is likely that AY057935 and AY057936 strains are, in fact, mutated clones of the respective parental strains, or that they were contaminated during laboratory procedures.

Alignment of the 37- and 33-bp sequences corresponding to the ISH probes revealed that they were 100% homologous with over 150 *H. pylori* strains but that there were at least two mismatches with different *Helicobacter* sp. such as *H. cetorum* and *H. bilis*. Interestingly, the ISH probes were also 100% homologous with several *Helicobacter* sp. isolates from horses, dogs, zoo seals, and other animals that live in close contact with humans.

### *In silico* verification of the specificity of the primers and probes

In order to determine whether the proposed method was specific for *H. pylori*, we performed a series of BLAST (bl2seq) of the sequence corresponding the RT-PCR primers and probe (71-bp of the 76 bp entire sequence) with the sequences of non-*H. pylori* bacteria. We observed the presence of 27 mismatches for *E. coli*, *S. bareilly*, and *S. flexneri*, 13 mismatches for *C. jejuni* and 6 mismatches for *H. heilmannii.*

### *In vitro* validation of the RT-PCR primers and probes

By real-time RT-PCR, pure cultures of *H. pylori* were positive whereas pure cultures of *E. coli* (Top10), *S. typhimurium* LT2, *V. cholerae* O139 (Classical Ogawa), *V. cholerae* O139 (El Tor), and *P. aeruginosa* were negative.

### *In vitro* validation of the *in situ* hybridization probe

**Figure 3:**
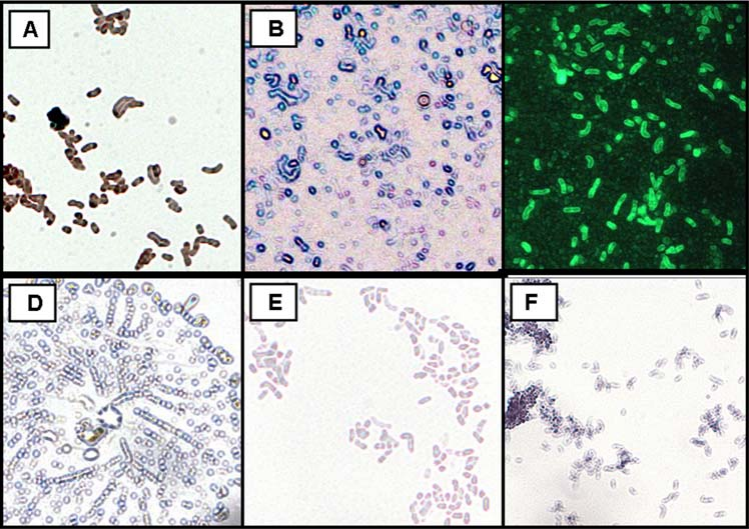
Smears of bacteria processed by ISH and FISH using biotin-labeled probe specific for *H. pylori 16S rRNA* (1,000X). *H. pylori* isolate processed by ISH and using biotin-labeled probe specific for *H. pylori 16S rRNA* (A; avidin peroxidase-DAB; brown; and B; avidin alkaline phosphatase BCIP/NBT; blue) or by FISH [C; avidin- fluorescein (FITC) stained green]. Negative controls (light violet stain due to the hematoxylin QS counterstaining but no brown or blue reaction): ISH using biotin-labeled probe specific for *H. pylori 16S rRNA* (avidin- peroxidase-DAB; negative) in a strain of *S. typhimurium* LT2 (D). Negative controls of methods using PBS (E) or scorpion toxin (F).

Pure cultures of *H. pylori* were positive whereas pure cultures of *E. coli*, *S. typhimurium*, *V. cholerae*, and *P. aeruginosa* were negative (Figure 3). This method is being used in the laboratory to specifically verify that *H. pylori* single colony isolates are not contaminated by other bacteria.

### Determination of *H. pylori* density in gastric biopsies from Rhesus monkeys by RT-PCR

Primary cultures of the first biopsy were negative in 105 monkeys that had less than 500 copies/100 ng of RNA extracted from the second biopsy. The number of positive cultures increased progressively with increasing *H. pylori* density (500–5,000: 2/29; 5,000–50,000: 8/30; and >50,000: 15/30).

### Visualization of *H. pylori* in Rhesus monkey gastric biopsies by Genta and ISH

**Figure 4:**
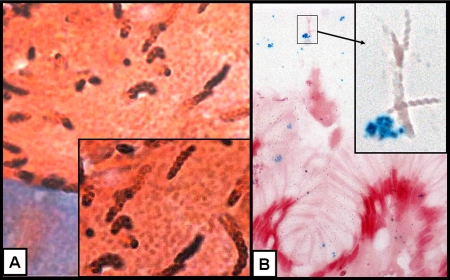
Gastric biopsy of a rhesus monkey with *H. pylori* and *H. heilmannii* co-infection. Genta stain (A: 400X; insert: 1,000X) demonstrates the presence of high *H. heilmannii* infection (typical tightly spiraled, ∼10 µm-long rods), in addition to a few *H. pylori*–like bacteria (∼3 µm-long and curved). *In situ* hybridization (ISH) with *16S rRNA* probe (B: 400X; insert: 1,000X) demonstrates the presence of *H. pylori* (stained blue by the avidin alkaline phosphatase (nitroblue tetrazolium) while other, tightly spiraled bacteria are negative.

Biopsies from a Rhesus monkey colonized by both *H. pylori* and *H. heilmannii* demonstrated that only *H. pylori*–shaped bacteria were detected by ISH (Figure 4).

## Discussion

In the present study, we used an *in silico* approach to demonstrate that a 546-bp domain of *H. pylori 16S rRNA* is highly conserved in most *H. pylori 16S rRNA* sequences registered in the NCBI GenBank and that a 229-bp sub-domain of this conserved region is specific to *H. pylori*. Within this sub-domain, it was possible to design an ISH probe and a set of real-time RT-PCR primers and a TaqMan probe that are 100% homologous with over 100 *H. pylori* strains isolated from humans residing in four continents, from monkeys [14], [32], and from cats [33]. In addition, 100% homology was found with many *Helicobacter* sp. that were later identified as *H. pylori*. Two are listed as *H. nemestrinae* (AF363064 and AF348617), although the strains are now recognized to be *H. pylori* [27]. The revised GenBank description of the strain, under “source” and “organism” reflects the correction, although the name *H. nemestrinae* still remains associated with the accession number. Four other strains are published in GenBank as *Helicobacter* sp. “liver” (AF142583 and AF142585) although a subsequent phylogenetic study suggested that they are, in fact, *H. pylori* [26]. Five other sequences correspond to those of *H. pylori*–like DNA extracted from the liver of patients with hepatitis C [31]. Another strain is currently listed as a *H. heilmannii* (AF506794) in NCBI, but this strain is not mentioned in the publication [34] because it clustered with *H. pylori* by both *16S rRNA* and urease sequencing (O'Rourke, personal communication). Finally, 13 of the 100% homologous *Helicobacter sp.* strains are extremely close to *H. pylori* and were isolated from carnivores including cheetahs and a tiger, and from horses. Interestingly, these animals live in close association with humans and they may be infected with *H. pylori* [29]. Importantly, the 76-bp region corresponding to the primers and probe and the 37- and 33-bp sequences of the ISH probes have multiple mismatches with non-*H. pylori* sequences.

*16S rRNA* was chosen for detection and quantification of *H. pylori* because ribosomal RNAs exhibit a high degree of functional and evolutionary homology within all bacteria and those sequences have been used for phylogenetic comparisons and classifications of microbial organisms [35], [36]. Analysis of *16S rRNA* in bacteria led to the detection of conserved, semi-conserved and non-conserved domains in this gene and to the development of molecular techniques that can specifically identify a variety of bacteria species [37]. *Helicobacter* genus-specific primers for *16S rRNA* have been used in PCR amplification as a screening tool to detect *Helicobacter* organisms in biological specimens [16], [38]. Although the sequences corresponding to these primers are common to most species within the genus *Helicobacter*, sequencing and restriction enzyme analysis showed that the nucleotide sequence delimited by the primers varies with the species [16], [38]. In order to specifically identify *H. pylori*, Ho et al. proposed an assay based on PCR amplification of a 109-nucleotide segment within the *16S rRNA* sequence [13], but these primers were subsequently shown to be non-specific for *H. pylori* [39].

In recent years, real-time RT-PCR and ISH have become standard methods in well-equipped laboratories and many well-trained laboratory technicians have the required expertise to perform the tests. Therefore, we believe that the information provided in the present paper will lead to their use in clinical practice, especially since the calculated cost for real-time RT-PCR reagents and supplies is less that $2.00/sample.

In summary, a 76-bp region of *H. pylori 16S rRNA* that is common to a large number of *H. pylori* sequences and is specific to this bacterium was used to design primers and probes to be used in real-time RT-PCR and ISH assays. Both approaches are very sensitive and specific for *H. pylori* and the real time RT-PCR assay can be used readily in most modern laboratories if frozen samples have been saved. If only archived specimens are available, then the more specialized *in situ* hybridization assay can be used. We propose that both assays combine sensitivity and specificity, making them strong clinical tools for precise and rapid identification of *H. pylori* in biological specimens harvested from humans, animals, or environmental source.

## Supporting Information

Methods S1 Text
(0.04 MB DOC)

## References

[1] Atherton JC (2006). The pathogenesis of Helicobacter pylori-induced gastro-duodenal diseases.. Annu Rev Pathol.

[2] Forman D, Goodman KJ (2000). The epidemiology of stomach cancer: correlating the past with the present. Socioeconomic influences in early life can influence mortality in adult life.. BMJ.

[3] Brenner H, Arndt V, Stegmaier C, Ziegler H, Rothenbacher D (2004). Is *Helicobacter pylori* infection a necessary condition for noncardia gastric cancer?. Am J Epidemiol.

[4] Amieva MR, El-Omar EM (2008). Host-bacterial interactions in *Helicobacter pylori* infection.. Gastroenterology.

[5] Semino-Mora C, Doi SQ, Marty A, Simko V, Carlstedt I (2003). Intracellular and interstitial expression of *H. pylori* virulence genes in gastric precancerous intestinal metaplasia and adenocarcinoma.. J Infect Dis.

[6] Dubois A, Boren T (2007). Helicobacter pylori is invasive and it may be a facultative intracellular organism.. Cell Microbiol.

[7] Necchi V, Candusso ME, Tava F, Lunetti O, Ventura U (2007). Intracellular, intercellular and stromal invasion of gastric mucosa, preneoplastic lesions, and cancer by H. pylori.. Gastroenterology.

[8] Aspholm M, Olfat FO, Norden J, Sonden B, Lundberg C (2006). SabA Is the *H. pylori* Hemagglutinin and Is Polymorphic in Binding to Sialylated Glycans.. PLoS Pathog.

[9] Camorlinga-Ponce M, Romo C, Gonzalez-Valencia G, Munoz O, Torres J (2004). Topographical localisation of *cagA* positive and *cagA* negative *Helicobacter pylori* strains in the gastric mucosa; an *in situ* hybridisation study.. J Clin Pathol.

[10] Kolbert CP, Persing DH (1999). Ribosomal DNA sequencing as a tool for identification of bacterial pathogens.. Curr Opin Microbiol.

[11] Smith SI, Oyedeji KS, Arigbabu AO, Cantet F, Megraud F (2004). Comparison of three PCR methods for detection of *Helicobacter pylori* DNA and detection of *cagA* gene in gastric biopsy specimens.. World J Gastroenterol.

[12] Gorkiewicz G, Feierl G, Schober C, Dieber F, Kofer J (2003). Species-specific identification of *Campylobacters* by partial 16S rRNA gene sequencing.. J Clin Microbiol.

[13] Ho SA, Hoyle JA, Lewis FA, Secker AD, Cross D (1991). Direct polymerase chain reaction test for detection of *Helicobacter pylori* in humans and animals.. J Clin Microbiol.

[14] Drazek ES, Dubois A, Holmes RK (1994). Characterization and presumptive identification of Helicobacter pylori isolates from rhesus monkeys.. J Clin Microbiol.

[15] Smith JG, Kong L, Abruzzo GK, Gill CJ, Flattery AM (1996). PCR detection of colonization by *Helicobacter pylori* in conventional, euthymic mice based on the 16s ribosomal gene sequence.. Clin Diagn Lab Immunol.

[16] Fox JG, Dewhirst FE, Shen Z, Feng Y, Taylor NS (1998). Hepatic *Helicobacter* species identified in bile and gallbladder tissue from Chileans with chronic cholecystitis.. Gastroenterology.

[17] Akopyants N, Bukanov NO, Westblom TU, Berg DE (1992). PCR-Based RFLP analysis of DNA sequence diversity in the gastric pathogen *Helicobacter pylori*.. Nucleic Acids Res.

[18] Eckloff BW, Podzorski RP, Kline BC, Cockerill FR3 (1994). A comparison of 16S ribosomal DNA sequences from five isolates of *Helicobacter pylori*.. Int J Syst Bacteriol.

[19] Tatusova TA, Madden TL (1999). BLAST 2 Sequences, a new tool for comparing protein and nucleotide sequences.. FEMS Microbiol Lett.

[20] Genta RM, Robason GO, Graham DY (1994). Simultaneous visualization of *Helicobacter pylor*i and gastric morphology: A new stain.. Human Pathology.

[21] Aspholm M, Kalia A, Ruhl S, Schedin S, Arnqvist A, Minoru Fukuda (2006). *Helicobacter pylori* adhesion to carbohydrates.. Methods in Enzymology.

[22] Lan Z-D, Dai L, Zhuo X-L, Feng J-C, Xu K (1999). Gene cloning and sequencing of BmK AS and BmK AS-1, two novel neurotoxins from the scorpion *Butus martensi* Karsch.. Toxicon.

[23] Applera Corporation (2001). Primer Express® Software v2.0. Applications-Based Primer Design Software: Applications Tutorials.. http://www.biotech.uiuc.edu/centers/Keck/Functional_genomics/taqman/Designing%20MGB%20probes%20and%20primers.pdf.

[24] Giulietti A, Overbergh L, Valckx D, Decallonne B, Bouillon R (2001). An overview of real-time quantitative PCR: applications to quantify cytokine gene expression.. Methods.

[25] Liu H, Semino-Mora C, Mog SR, Boren T, Dubois A (2006). Effect of a carcinogen on *H. pylori* infection: Expression of cytokines and TGFâ Receptor 2 in rhesus monkeys.. Gastroenterology.

[26] Avenaud P, Marais A, Monteiro L, Le Bail B, Bioulac SP (2000). Detection of *Helicobacter* species in the liver of patients with and without primary liver carcinoma.. Cancer.

[27] Suerbaum S, Kraft C, Dewhirst FE, Fox JG (2002). *Helicobacter nemestrinae* ATCC 49396T is a strain of *Helicobacter pylori* (Marshall et al. 1985) Goodwin et al. 1989, and *Helicobacter nemestrinae* Bronsdon et al. 1991 is therefore a junior heterotypic synonym of *Helicobacter pylori*.. Int J Syst Evol Microbiol.

[28] Sturegard E, Hertervig E, Sjunnesson H, Wadstrom T (2004). *Helicobacter* species in human colon biopsies.. Aliment Pharmacol Ther.

[29] Eppinger M, Baar C, Linz B, Raddatz G, Lanz C (2006). Who ate whom? Adaptive *Helicobacter* genomic changes that accompanied a host jump from early humans to large felines.. PLoS Genet.

[30] Contreras M, Morales A, Garcia-Amado MA, DeVera M, Bermudez V (2007). Detection of Helicobacter-like DNA in the gastric mucosa of thoroughbred horses.. Lett Appl Microbiol.

[31] Castera L, Pedeboscq A, Rocha M, Le BB, Asencio C (2006). Relationship between the severity of hepatitis C virus-related liver disease and the presence of *Helicobacter* species in the liver: A prospective study.. World J Gastroenterol.

[32] Doi SQ, Kimbason T, Reindel J, Dubois A (2005). Molecular characterization of *Helicobacter pylori* strains isolated from cynomolgus monkeys (M. fascicularis).. Vet Microbiol.

[33] Handt LK, Fox JG, Dewhirst FE, Fraser GJ, Paster BJ (1994). *Helicobacter pylori* isolated from the domestic cat: Public health implications.. Infect Immun.

[34] O'Rourke JL, Solnick JV, Neilan BA, Seidel K, Hayter R (2004). Description of ‘Candidatus *Helicobacter heilmannii*’ based on DNA sequence analysis of 16S rRNA and urease genes.. Int J Syst Evol Microbiol.

[35] Drancourt M, Bollet C, Carlioz A, Martelin R, Gayral JP (2000). 16S ribosomal DNA sequence analysis of a large collection of environmental and clinical unidentifiable bacterial isolates.. J Clin Microbiol.

[36] Gorkiewicz G, Feierl G, Schober C, Dieber F, Kofer J (2003). Species-specific identification of *Campylobacters* by partial 16S rRNA gene sequencing.. J Clin Microbiol.

[37] Gray MW, Sankoff D, Cedergren RJ (1984). On the evolutionary descent of organisms and organelles: a global phylogeny based on a highly conserved structural core in small subunit ribosomal RNA.. Nucleic Acids Res.

[38] Riley LK, Franklin CL, Hook RR, Besch-Williford C (1996). Identification of murine *Helicobacters* by PCR and restriction enzyme analyses.. J Clin Microbiol.

[39] Chong SK, Lou Q, Fitzgerald JF, Lee CH (1996). Evaluation of 16S rRNA gene PCR with primers Hp1 and Hp2 for detection of *Helicobacter pylori*.. J Clin Microbiol.

