# Supplementary material for: Specific and Sensitive Detection of H. pylori in Biological Specimens by Real-Time RT-PCR and In Situ Hybridization
Source: PLoS One. 2008 Jul 16;3(7):e2689. doi: 10.1371/journal.pone.0002689 (PMC2481290; doi:10.1371/journal.pone.0002689)
Supplement: Methods S1 — Text (0.04 MB DOC) [file pone.0002689.s001.doc]

**Methods S1**

**Random Amplification of Polymorphic DNA (RAPD)**. *H. pylori* genomic DNA was PCR-amplified in 25 μl reaction mix containing 12.5 ul Premix Ex-Taq (Takara Bio Inc.), 20 pmoles of primer and 200 ng of template DNA. The parameters set in a Perkin Elmer thermocycler 2400 (PE Applied Biosystems, Foster City) were: [94^o^C, 5 min; 36^o^C, 5 min; and 72^o^C, 5 min], 4 cycles, followed by 30 cycles of [94^o^C, 1 min; 36^o^C, 1 min; and 72^o^C, 2 min], and finally 72^o^C for 10 min. PCR products were resolved by electrophoresis in ethidium bromide-stained 2% agarose gels.

***16S rRNA* gene amplification and sequencing.** Total DNA was extracted from each patient’s isolate and PCR-amplified using published primers [1]. Amplification was performed at annealing temperature of 50^o^C for 35 cycles using a Perkin Elmer 2400 thermocycler. The amplified segment was resolved by electrophoresis on ethidium bromide stained-agarose gel and visualized by UV transillumination. The DNA band was cut out from the agarose gel and purified using MinElute^TM^ Gel extraction kit (Qiagen). The sequence of the purified *16S rRNA* gene was determined using sets of overlapping primers [1], a dye terminator reagent and an automated DNA sequencer (PE Applied Biosystems, Foster City).

***In situ* hybridization**

1. Pre-hybridization: Sections were deparaffinized, rehydrated in grades of ethanol, and treated with proteinase K (Roche Diagnostic, Indianapolis) for unmasking nucleic acids and preparation for probe penetration. Sections were treated with hybridization mixture and incubated with bovine serum albumin (BSA) to block non-specific binding.

2. Hybridization: 50-100 μl of the probes denatured by heating for 10 min at 65ºC were placed on the tissue and incubated for 18 hrs at 37ºC.

3. Post-hybridization: unbound probe was removed by successive washes

4. Detection of *H. pylori* Gene Expression. Sections were incubated with streptavidin conjugated alkaline phosphatase (AP; Roche Diagnostic, Indianapolis) or avidin-labeled peroxidase to detect the biotin-labeled *16S rRNA* probe.

Unbound antibody-alkaline phosphatase peroxidase were washed away, bound alkaline phosphatase or peroxidase was detected using a chromogenic substrate (nitroblue tetrazolium (BCIP/NBT) or 3,3’ diaminobenzidine (DAB) respectively), and slides were counterstained (Nuclear Fast Red or hematoxylin QS for alkaline phosphatase or peroxidase detection use respectively), washed, dehydrated, cleared in xylene, and mounted with Permount (Biomedia Corporation, Foster City).

For fluorescence ISH (FISH), a similar method was used [labeling of *H. pylori 16S rRNA* probe was labeled with biotin, detection with avidin-fluorescein isothiocyanate (FITC), mounting with Vectashield (Vector), resulting in bright green reaction] [2,3].

Reference List

1. Eckloff BW, Podzorski RP, Kline BC, Cockerill FR3 (1994) A comparison of 16S ribosomal DNA sequences from five isolates of *Helicobacter pylori*. Int J Syst Bacteriol 44: 320-323.

2. Semino-Mora C, Doi SQ, Marty A, Simko V, Carlstedt I, Dubois A (2003) Intracellular and interstitial expression of *H. pylori* virulence genes in gastric precancerous intestinal metaplasia and adenocarcinoma. J Infect Dis 187: 1165-1177.

3. Aspholm M, Kalia A, Ruhl S, Schedin S, Arnqvist A, et al. (2006) *Helicobacter pylori* adhesion to carbohydrates. In: Minoru Fukuda, editors. Methods in Enzymology. New York: Elsevier. pp. 293-239.
